# Colorectal Cancer Biomarkers: Where Are We Now?

**DOI:** 10.1155/2015/149014

**Published:** 2015-05-27

**Authors:** Maria Gonzalez-Pons, Marcia Cruz-Correa

**Affiliations:** ^1^Department of Gastrointestinal Oncology and Genetics, University of Puerto Rico Comprehensive Cancer Center, San Juan, PR 00927-6346, USA; ^2^UPR-MDACC Partnership for Excellence in Cancer Research Program, University of Puerto Rico Medical Sciences Campus, San Juan, PR 00936-5067, USA; ^3^Department of Medicine, Biochemistry, and Surgery, University of Puerto Rico School of Medicine, San Juan, PR 00936-5067, USA

## Abstract

Colorectal cancer is one of the major causes of cancer-related death in the Western world. Patient survival is highly dependent on the tumor stage at the time of diagnosis. Reduced sensitivity to chemotherapy is still a major obstacle in effective treatment of advanced disease. Due to the fact that colorectal cancer is mostly asymptomatic until it progresses to advanced stages, the implementation of screening programs aimed at early detection is essential to reduce incidence and mortality rates. Current screening and diagnostic methods range from semi-invasive procedures such as colonoscopy to noninvasive stool-based tests. The combination of the absence of symptoms, the semi-invasive nature of currently used methods, and the suboptimal accuracy of fecal blood tests results in colorectal cancer diagnosis at advanced stages in a significant number of individuals. Alterations in gene expression leading to colorectal carcinogenesis are reflected in dysregulated levels of nucleic acids and proteins, which can be used for the development of novel, minimally invasive molecular biomarkers. The purpose of this review is to discuss the commercially available colorectal cancer molecular diagnostic methods as well as to highlight some of the new candidate predictive and prognostic molecular markers for tumor, stool, and blood samples.

## 1. Introduction

In the United States (US), it is estimated that there are more than a million people currently living with colorectal cancer [1]. Unlike many other malignancies, colorectal cancer (CRC) is a preventable and potentially curable disease if high-risk adenomas and early stage tumors are removed. Patient survival is highly dependent on the tumor stage at the time of diagnosis. Only 40% of CRC cases are diagnosed at localized stages in the US [2]. The overall 5-year survival of CRC patients is close to 65%; 5-year survival rates range from 90% for patients with localized disease to 70% and 13% for regional and distant stages, respectively [2]. Due to the fact that CRC is mostly asymptomatic until it progresses to advanced stages, the implementation of screening programs aimed at early detection is essential to reduce incidence and mortality rates.

Advances in molecular biology in the last three decades have helped elucidate some of the genetic mechanisms leading to colorectal carcinogenesis. Most CRC cases are due to sporadic genetic and/or epigenetic changes, but up to 10–20% of all CRC cases have a familial component [3, 4]. Sporadic colorectal carcinogenesis is a result of complex multifactorial processes resulting in the alteration of normal colon epithelial cell cycle. Therefore, the substantial genetic heterogeneity in colorectal tumors has to be taken into account when developing novel molecular diagnostic methods since they may display features of multiple affected cellular pathways. There are three major molecular mechanisms that cause aberrant gene expression resulting in colon carcinogenesis: microsatellite instability (MSI), chromosomal instability (CIN), and the CpG island methylator phenotype (CIMP) (Table 1). These pathways lead to a transition in lesion pathology and progression to malignancy, which is accompanied by deregulated gene expression of tumor suppressor genes and oncogenes. These cytogenetic alterations have been considered as potential CRC molecular markers because they can provide the clinician with diagnostic, prognostic, and predictive treatment response information.

Although some of the CRC screening tests available have proven to be effective in the reduction of incidence and mortality, a highly specific, noninvasive detection method has yet to be discovered. The American Cancer Society recommends various screening tests ranging from semi-invasive procedures, such as colonoscopy, to noninvasive stool tests (Table 2). Nevertheless, the combination of the absence of symptoms, the semi-invasive nature of endoscopic methods, and the suboptimal accuracy of fecal blood tests results in diagnosis at later stages in a significant number of individuals. Molecular tests are expected to be more sensitive and specific than current methods. They will also provide genetic information about the malignancy in progression. Intense research efforts aiming at identifying molecular markers (DNA, RNA, or protein) to develop novel, noninvasive biomarker detection methods for CRC in blood and stool are underway.

The ideal molecular marker should have the following qualities: it should have high sensitivity and specificity; it should be safe and affordable so that it can be broadly accepted by patients; it should be easy to measure; and it should be consistently detected across genders and ethnic groups [5–8]. A robust biomarker should detect genomic alterations and/or variations in protein expression that specifically correlate to the disease helping clinicians make an accurate diagnosis. Molecular markers can also be used to assess the risk of future disease, the aggressiveness of the malignancy over the time, and the probability that a patient will respond to a particular treatment, thereby helping the clinician make personalized treatment decisions. New molecular CRC detection methods are currently being evaluated, but most of these still have to be validated in large, randomized trials prior to implementation in the clinic. In this review, we will outline the currently available and developing tumor, stool, and blood sporadic CRC molecular diagnostic, predictive, prognostic, and surveillance biomarkers.

*Glossary*. Types of molecular markers:

 Diagnostic: used for risk stratification and early detection.
 Prognostic: gives an indication of the likely progression of the disease.
 Predictive: predicts treatment response.
 Surveillance: used to monitor disease recurrence.

## 2. Tumor Biomarkers

Colorectal tumors can be classified according to molecular markers. The current conventional molecular tests used when evaluating CRC patients include microsatellite instability (MSI) analysis and* BRAF *and* KRAS *mutation analysis. The presence of MSI and/or certain mutations or epimutations in colorectal tumors provides clinicians with the information needed to choose the appropriate treatment. Due to the high variability of clinical responses to CRC treatment, there is a need to identify novel predictive and prognostic molecular classifiers to make the best treatment decisions for the patient taking into account their prognosis as well as their predicted response to chemotherapeutics.

### 2.1. Currently Available Tumor-Based Tests

#### 2.1.1. Microsatellite Instability (MSI)

Mutations and/or epimutations in genes involved in the DNA mismatch repair system (MMR),* MLH1, MSH2, MSH6,* and* PMS2*, result in alterations in highly repeated DNA sequences (microsatellites). MSI is a hallmark of Lynch Syndrome, an inherited CRC syndrome, and is used as a diagnostic marker for this disease. In sporadic CRC, 10–15% of tumors display MSI although somatic mutations in MMR genes are rarely found. Methylation induced silencing of* MLH1* is responsible for the majority of sporadic CRC with MSI [9].

A panel of five mononucleotide markers (Bat-25, Bat-26, NR-21, NR-24, and MONO-27) is currently being used by most clinical laboratories to detect MSI. The definition of MSI (also known as MSI-high) is based on having ≥30% of unstable loci using monocleotide and dinucleotide markers; tumors with 10–29% of unstable loci in the panel are considered MSI-low. Absence of expression of the MMR proteins in tumor tissue is also used as a surrogate test indicative of MSI. Sporadic CRC tumors with MSI are mostly located in the proximal colon, present with mucinous or signet ring histology, are poorly differentiated, have an abundance of tumor infiltrating lymphocytes, and have* BRAF *mutations [10–12]. There is still controversy regarding if MSI-low tumors are a CRC subtype.

Accumulating evidence suggests that MSI status may be a useful prognostic and predictive sporadic CRC marker. MSI is associated with increased patient survival and a favorable prognosis. Data from retrospective studies, population studies, and meta-analyses support that patients with CRC tumors with MSI have better outcomes than patients with microsatellite stable tumors. In a population-based study with 607 patients, the patients that had MSI had a more favorable prognosis and decreased likelihood of lymph node and systemic metastasis [13, 14]. Additional studies support that MSI may be an independent predictor of less aggressive disease and better outcomes [14–16]; however, the use of MSI as prognosis biomarker has not yet been implemented in the clinic. Although controversial, several studies have reported an association between MSI and the development of additional colorectal carcinomas in sporadic CRC patients suggesting that MSI status may also be useful as a predictor of the risk of developing metachronous CRC [17, 18]. Additional studies are needed to provide solid evidence supporting that sporadic CRC patient management should take MSI status into consideration.

Accumulating evidence supports that MSI status may predict responsiveness to adjuvant chemotherapy. Reports from clinical trials, retrospective case series, and meta-analysis have reported that patients with MSI tumors do not benefit from 5-fluorouracil (5-FU) adjuvant chemotherapy compared to patients with microsatellite stable tumors (MSS) [16, 19–22]. In a meta-analysis based on studies stratifying patient's overall survival by 5-FU therapy, survival data in patients with colorectal tumors with MSI was heterogenous compared to individuals with MSS tumors. A significant improved overall survival was only found in patients with MSS tumors following 5-FU therapy [21]. In addition, some studies have also proposed MSI status as a predictive marker for response to irinotecan [23, 24]. The value of using MSI as a predictive marker for chemosensitivity remains controversial and is still under evaluation.

#### 2.1.2. *KRAS* Gene Mutations

Mutations in genes associated with chemoresistance to particular compounds are currently used as predictive markers in CRC in order to identify the best treatment regime for patients. Detection of* KRAS *mutations is currently the most utilized predictive marker for response to the anti-EGFR (epidermal growth factor receptor) antibody-based therapies, cetuximab and panitumumab [25]. However, recent studies have reported compelling evidence that, in addition to KRAS, mutations in NRAS predict nonresponse to anti-EGFR therapy [26, 27]. These studies support the use of extended RAS (KRAS and NRAS) mutational analyses as negative predictive markers for anti-EGFR therapy in metastatic CRC (mCRC) [28].

The* KRAS* protooncogene encodes a small G protein (guanosine triphosphate/guanosine diphosphate binding protein) downstream of EGFR in the PI3K/PTEN/AKT and RAF/MEK/ERK signaling pathways. Most of the activating mutations, approximately 90%, are found in codons 12 and 13 of exon 1. Close to 5% of the mutations are found in codon 61 in exon 2 [11, 29].* NRAS *(*neuroblastoma RAS viral oncogene homolog*), a gene closely related to* KRAS, *is mutated in 3–5% of CRC cases, mostly in codon 61. It has been associated with poor responses to cetuximab and panitumumab-FOLFOX4 [26, 30, 31].

Activating* KRAS *mutations have been significantly associated with the lack of response to anti-EGFR therapies in patients with mCRC [25, 32, 33]. Tumors with* KRAS *mutations have response rates to cetuximab and panitumumab ranging from 26-41% and from 11–17%, respectively. Approximately 60–70% mCRC patients with wild type* KRAS *have a limited response to EGFR antibody-based therapy, which suggests that additional mutations may contribute to anti-EGFR treatment response [34]. Clinical guidelines established by the National Comprehensive Cancer Network and the American Society of Clinical Oncology recommend* KRAS *mutational analysis for mCRC patients prior to the use of cetuximab and panitumumab. Furthermore, there are reports supporting that mutations in codons 12 and 13 are associated with worse prognosis and poor survival in mCRC patients [35, 36]. However, it has not been clearly established if the worse prognosis associated with* KRAS* mutations is independent of the treatment regime used. The use of* KRAS* as a predictive biomarker for response to anti-EGFR therapies is the standard of care in mCRC patients [37] and the first instance of personalized medicine for these patients.

#### 2.1.3. *BRAF* Gene

*BRAF*, a* RAF* gene family serine/threonine kinase, is the immediate downstream effector of KRAS in the Ras/Raf/MAPK signaling pathway. Mutations in the* BRAF *gene have been associated with CRC development [38, 39] and are present in 40–50% of sporadic MSI-high CRC [40–42]. These are absent in Lynch syndrome patients, making* BRAF* mutation status a very useful diagnostic tool to distinguish between familial and sporadic CRC. A missense mutation resulting in a valine to glutamic acid substitution (V600E) is the most common mutation observed [43].* KRAS *and* BRAF *mutations are generally mutually exclusive in colorectal tumors [44]. Recent studies suggest that* BRAF *mutations may also be used as predictive markers for* EGFR*-targeted therapy [31, 44–48]. Mutations in* BRAF *are associated with poor prognosis; however, it is still unclear if this association is independent of treatment. The National Comprehensive Cancer Network now recommends that in cases with wild type* KRAS, BRAF* testing should be considered prior to deciding which treatment strategy is optimal for the patient.

### 2.2. Innovative Tumor-Based Tests

#### 2.2.1. CpG Island Methylator Phenotype

The molecular classification of tumors is evolving as we gain a comprehensive knowledge about the mechanisms and processes resulting in colorectal carcinogenesis. The epimutation status of tumors has gained importance since the discovery that methylation-driven transcriptional regulation leads to colorectal carcinogenesis and that the CpG island methylator phenotype (CIMP) status correlates to a particular CRC subtype. CIMP high colorectal tumors are more prevalent in women and are associated with* BRAF *mutations. They display distinct characteristics which include: proximal tumor location, poor differentiation, mucinous histology, MSI, and low frequency of* TP53 *mutations [49–53]. Currently, the panel of methylation markers used to define CIMP and the methylation detection technique has not been standardized. The lack of a consensus panel has resulted in controversial reports. Larger studies using a consensus panel and sensitive, methylation detection techniques will resolve the discrepancies in this field and will likely yield a CRC-specific methylation signature that could be developed into a diagnostic tool in the future.

#### 2.2.2. RNA Expression

Gene expression analyses between tumor and normal tissue have contributed to a better understanding on the interplay between overexpressed or underexpressed genes and the affected pathways resulting in colorectal neoplasms. These efforts have resulted in a wealth of publicly available RNA expression data used to identify differentially expressed transcripts, which can be used to identify a CRC-specific signature. Expressed sequence tags (EST), serial analysis of gene expression (SAGE), and microarray data have identified numerous promising candidate tumor biomarkers. Validation of a 23-gene microarray-based prognostic signature proposed by Wang et al. [77] reported a 67% relapse predictive value [78]. A seven-gene panel based on this study was tested in a larger study with a better performance, but a robust multigene signature has not yet been defined [79]. Efforts are underway to identify RNA gene expression signature from paraffin-embedded tumor tissue via real-time PCR, which will be validated in a cohort of 2000 patients (QUASAR trial) [80].

#### 2.2.3. MicroRNAs

Accumulating evidence supporting that noncoding microRNAs (miRNAs) contribute to oncogenesis have resulted in multiple studies aiming at identifying a miRNA biomarker panel. Studies examining miRNA expression in CRC have shown a total of 362 differentially expressed miRNAs when compared to noncancerous tissue; 242 were upregulated and 120 were downregulated [81]. The use of different platforms for miRNA expression profiling has resulted in contradictory reports; nevertheless, 101 of the 362 miRNAs were consistently reported to be dysregulated in CRC. Further clinical and mechanistic studies are needed to elucidate the clinical utility of miRNAs (individual or panels of miRNAs) as diagnostic, prognostic, and/or possible therapeutic tools.

#### 2.2.4. EGFR Pathway

Beyond* KRAS* testing, other studies have focused on identifying additional biomarkers to predict response to anti-EGFR treatment in patients with wild type* KRAS* (up to 70% are unresponsive to cetuximab or panitumumab [34]). Expression of the EGFR ligands* amphiregulin *and* epiregulin* have been associated with increased response to cetuximab [82, 83]. These ligands are being explored as candidate predictive markers. A four-gene expression-based biomarker panel including these ligands in addition to* DUSP6 *(*dual specificity phosphatase-6*) and* SLC26A3 *(*Solute carrier family 26, member 3*) has been shown to predict response to cetuximab in patients with wild type* KRAS *[84]. EGFR signaling triggers two main intracellular cascades, one involving KRAS and BRAF leading to the activation of mitogen activated kinases and another resulting in the phosphorylation of AKT1 via interactions between PIK3CA and PTEN. Mutations in both* PIK3CA* and* PTEN* have also been evaluated as predictive markers for anti-EGFR therapies. Mutations within* PIK3CA* have been found to independently affect the response to both cetuximab and panitumumab. This gene is mutated in approximately 20% of CRC patients [85] with most mutations occurring in exon 9 and exon 20 [86]. Mutation analysis of* PIK3CA* has been proposed as predictive marker in combination with* KRAS* mutation testing prior to administering anti-EGFR therapy. In addition, patients with* PIK3CA* mutations have been reported to have worse clinical outcomes in terms of progression free survival [48]. Loss of* PTEN* expression occurs in approximately 30% of sporadic CRC cases and has been associated with unresponsiveness to cetuximab [87] and with worse overall survival in patients with mCRC [48]. Future studies will undoubtedly identify additional predictive biomarkers to enhance and guide treatment strategies for CRC patients.

## 3. Stool Biomarkers

The rationale behind stool-based molecular diagnostic tests for CRC is based on the mechanisms leading to the presence of these markers in stool. The presence of tumor markers in stool can be attributed to leakage, exfoliation, or secretion [88]. The disturbance of blood or vessels by tumor growth results in the leakage of markers into the colon lumen. This process may not be continuous and also occurs in nonneoplastic lesions. As a result, leaked markers have limited sensitivity and specificity. Exfoliated and secreted markers come from colonocytes, both vital and apoptotic, shed in the colorectal lumen. These types of stool markers are assumed to be highly specific because they are directly derived from the tumor. Although considerable efforts are being made to identify DNA, RNA, and protein markers present in stool to develop novel detection methods, only three clinically stool-based tests for CRC diagnosis are currently available: gFOBT, fecal immunochemical test (FIT), and detection of vimentin methylation.

### 3.1. Currently Available Stool-Based Tests

#### 3.1.1. Fecal Blood Tests

The gFOBTs are based on the detection of the pseudoperoxidase activity of heme in stool samples resulting from bleeding in adenomatous or neoplastic lesions, therefore detecting the presence of occult blood. The most commonly used gFOBTs are the guaiac-infused Hemoccult II and Hemoccult II SENSA (which has improved sensitivity) (Beckman Coulter, Brea, CA). One of the disadvantages of this method is that since colorectal bleeding might be intermittent, this test has to be performed on multiple occasions in order for it to be sensitive. In addition, gFOBTs are not specific for human pseudoperoxidase and may detect bleeding from any site in the gastrointestinal tract. Prior to testing, patients need to adhere to a three-day diet that eliminates meats and NSAIDs because ingestion of certain foods and drugs may cause false-positive results. Nevertheless, randomized controlled clinical trials in the United States, England, and Denmark have shown that gFOBT testing once or twice a year significantly reduces CRC mortality [89–92].

An alternative to the gFOBT is the FIT, which has higher sensitivity and an improved detection rate for advanced neoplasia [93, 94]. This test uses monoclonal or polyclonal antibodies to detect the globin in human hemoglobin using an enzyme-linked immunosorbent assay (ELISA) [95]. The FIT is specific to bleeding from the distal gastrointestinal tract due to the fact that globin is gradually degraded as it passes through the intestine. Two types of FIT have been developed: a qualitative assay requiring visual interpretation and a quantitative test, which is analyzed automatically and determines the amount of hemoglobin present in the sample. Quantitative FITs, such as the OC-Sensa Micro (Eiken Chemical, Tokyo, Japan; sold in the United States by Polymedco, Cortland Manor, NY) and Insure (Enterix Inc., Quest Diagnostics Incorporated, Edison, NJ), have an advantage over qualitative assays because they eliminate observer variations in the interpretation of the results and have definitive cut-off levels improving reproducibility. Despite the advances made with fecal blood tests, they still have low sensitivity for detecting CRC and adenomas due in part to the fact that other gastrointestinal nonneoplastic conditions also cause bleeding and that not all adenomas or cancers bleed.

#### 3.1.2. DNA-Based Tests

The detection of CRC-specific DNA markers in stool has been studied extensively. These types of markers should have higher specificity since they are directly derived from tumor cells. However, the only stool DNA test that is commercially available in the US is ColoSure (Laboratory Corporation of America, https://www.labcorp.com). This test is based on the detection of* vimentin *methylation, which is found in 53–83% of colorectal tumors [96, 97]. The performance of this marker in detecting advanced adenomas has not yet been defined, but the sensitivity and sensitivity for colorectal cancer range from 72.5–83% and 53–86.9%, respectively [70].

### 3.2. Innovative Stool-Based Tests

#### 3.2.1. DNA-Based Tests

Shedding of neoplastic colonocytes is known to be higher than the shedding of normal healthy colonic cells in stool; therefore, CRC-specific biomarkers should have high specificity. Technical advances in the field, including improvements in the buffers used prior to analysis and the detection methods employed, have propelled research in this field which began in the early 1990s. Publications reporting the detection of tumor DNA in stool emerged in 1992 when* KRAS* mutations were detected in samples from CRC patients [98]. Mutations in other key genes implicated in CRC, such as* TP53 *and* APC, *were also detected, but the detection of a single or a combination of mutation markers did not achieve the specificity and sensitivity needed for a screening method. Due to tumor heterogeneity and the large amount of genes found to be mutated in CRC [99], a screening stool test solely based on mutation biomarkers would require testing a large number of genes. However, the detection of mutations in key genes may complement panels including other types of biomarkers.

Aberrantly methylated tumor-derived genes have been studied as CRC diagnostic markers. Changes in methylation occur early in carcinogenesis, appear to be stable, and can be feasibly detected in both stool and blood-based samples, making these markers ideal candidates for a noninvasive test for early detection of CRC. Single as well as combinations of methylation markers have been evaluated but have failed to reach high levels of specificity and sensitivity needed for clinical implementation. Recently, Imperiale et al. reported that in a large study including 9989 participants, a multitarget stool DNA test had higher sensitivity for the detection of advanced adenomas (42.4%) and CRC (92.3%) than FITs [69]. This cross-sectional study evaluated the screening potential of a multitarget stool test that detected* KRAS* mutations, methylation of* NDRG4* and* BMP3*, quantification of *β-actin* as a reference gene for DNA quantity, and immunochemical detection of hemoglobin. Although more false positives were detected using this panel, the screening value of this test is promising because it was able to detect polyps, including serrated adenomas, with high-grade dysplasia with a higher rate of detection than FITs. Serrated adenomas are the precursor lesions leading to serrated adenocarcinomas, a colorectal tumor subset that accounts for about 10% of all CRCs. Serrated adenocarcinomas are associated with* BRAF *mutations*, MLH1 *methylation, and CIMP [100]. The detection rates for serrated polyps and polyps with high-grade dysplasia using this multitarget stool test were 42.4% and 69.2% versus 5.1% and 46.2% using FIT, respectively. Further studies are warranted to determine the diagnostic accuracy, to reduce the number of false positives, and to determine adequate testing intervals and costs, among other factors, that may affect patient compliance to future CRC screening using this method.

#### 3.2.2. RNA-Based Tests

The detection of RNA markers in stool has not been as extensively studied as DNA biomarkers partly due to the fact that RNA is less stable than DNA in stool. Technological advances in RNA preservation buffers have made it feasible to study CRC tumor-specific RNA transcripts as stool biomarkers. Detection of single and combinations of tumor mRNA transcripts, such as* PTGS2 *and* MMP7, *have yielded high specificity for CRC [101]. Ongoing research in colorectal tumor gene expression profiles (transcriptomics) and noncoding RNA expression profiles (such as miRNAs) are currently being evaluated to identify candidate transcripts and explore their possible applications as CRC detection tools. Recently, Link et al. showed high reproducibility of miRNA extraction and expression analyses in stool samples where miR-21 and miR-106a were found to have higher expression in patients with adenomas or CRC when compared to individuals free of neoplasia [102].

#### 3.2.3. Stool Protein Detection

A novel method based on the detection of tumor-derived proteins would greatly improve the specificity of fecal CRC detection. Although the protein markers tested to date have mostly been detected in blood samples, proteins in stool such as calprotectin, CEA, and DAF, among others, have also been evaluated for their diagnostic potential. Thus far, the majority of the proteins studied failed to achieve the specificity and sensitivity required for CRC screening. Calprotectin was considered a very promising CRC marker, but like many of the other proteins evaluated, it was detected in stool from patients with inflammatory bowel disease (IBD) with a higher sensitivity and specificity [103]. Studies using haptoglobin, an acute-phase response protein, as a stool biomarker marker also showed encouraging results. CRC could be detected with a sensitivity of 92% and specificity of 98% with increasing sensitivity of 100% when combined with occult blood testing [104]. However, false positives may arise due to the fact that haptoglobin expression is mediated by inflammatory stimuli [105].

## 4. Blood Biomarkers

Extensive efforts have been made to identify blood-based markers for the early detection of CRC. Most of the candidate markers have been evaluated in clinical settings and are mostly detected in advanced stages. The availability of a blood-based, noninvasive test promises to improve screening compliance and to reduce the morbidity and mortality associated with this malignancy. To date, the most widely used blood-based CRC molecular marker is carcinoembryonic antigen (CEA), which has only proved to be valuable as a patient monitoring tool.

### 4.1. Currently Available Blood-Based Tests

#### 4.1.1. Carcinoembryogenic Antigen

Extensive research has been performed to identify CRC-specific antigens in blood. However, there only two blood-based biomarkers available to monitor CRC patients, CEA and carbohydrate antigen 19-9 (CA19-9). CEA, a high molecular weight glycoprotein, is found in embryonic tissue and colorectal malignancies. It was discovered in 1965 and is the only acceptable tumor marker to monitor CRC recurrence to date. Elevated CEA levels are considered a poor prognostic factor for resectable CRC and correlate with cancer progression [106]. Using this marker, the sensitivity increases according to tumor stage [107]; CEA levels decrease after tumor resection. However, high levels in blood are not specific for CRC and may be due to other diseases such as IBD, liver disease, pancreatitis, and other malignancies. Moreover, detection of this serum antigen marker is not an effective method for CRC screening because elevated levels of CEA are only detected in advanced stages of a fraction of all CRC patients. The CA19-9 antigen, compared to CEA, is less sensitive and specific for CRC. However, it is the best marker available to detect pancreaticobiliary malignancies. Therefore, CEA is still the antigen of choice to use as a prognostic marker after diagnosis and to monitor disease progression.

### 4.2. Innovative Blood-Based Tests

Prognosis for patients with advanced CRC is poor. Early detection is a key to reduce the morbidity and mortality associated with this disease. The availability of a noninvasive, blood-based CRC biomarker has the potential to increase patient adherence to CRC screening thereby increasing the number of tumors detected at earlier, more treatable stages. Technological advances in combination with a more comprehensive understanding of the molecular mechanisms contributing to colorectal carcinogenesis, have fueled intense efforts to identify CRC biomarkers detectable in blood. Circulating nucleic acids, proteins, and tumor cells have been evaluated as CRC diagnostic tools with promising levels of sensitivity and specificity. However, a new blood-based biomarker for CRC has not yet been implemented in the clinic.

#### 4.2.1. Cell-Free Nucleic Acids

The origin of cell-free nucleic acids in circulation is less well-defined than in stool. The release of DNA, RNA, and noncoding RNAs in cancer patients into circulation is attributed to a combination of tumor cell necrosis, apoptosis, and possibly secretion. Changes in the concentration and detection of tumor-specific alterations in DNA and/or dysregulated RNA expression profiles have been proposed as CRC-specific biomarkers. The development of diagnostic methods based on the detection of tumor-specific alterations in circulating DNA and/or RNA expression is particularly appealing because they can provide valuable molecular information about the tumor that may be used for diagnostic, predictive, and prognostic purposes.

Extracellular DNA in blood can be detected in the plasma or serum of patients suffering from a variety of diseases (including cancer) as well as in healthy individuals. Higher levels of cell-free DNA (cfDNA) in plasma or serum are generally found in cancer patients. This fact has led to intense research efforts focused on developing strategies to take advantage of this source of analyzable tumor-derived DNA [108]. Based on the premise that CRC patients have higher levels of cfDNA in blood, Frattini et al. examined the quantification of cfDNA as both a screening and surveillance tool. Prior to surgery, CRC patients had 25 times higher levels of cfDNA in plasma than healthy donors; cfDNA levels decreased after tumor resection while they increased in those with recurrence or metastasis [109]. In contrast, elevated CEA values were detected in only 37% of the cases.

#### 4.2.2. Integrity of Cell-Free DNA

The cfDNA strand integrity has also been explored as a CRC biomarker. Presumably, cfDNA released from necrotic cancer cells varies in size and is not uniformly truncated like cfDNA resulting from apoptosis. A small study reported that CRC patients had a significant increase in serum DNA integrity when compared to healthy individuals [110]. Although reports have highlighted the potential usefulness of cfDNA concentration and integrity as CRC biomarkers, the main limitation is that these changes in cfDNA are not specific for CRC.

#### 4.2.3. Genetic and Epigenetic Alterations in Circulation

Detection of tumor-derived genetic and epigenetic alterations in blood samples has been explored as candidate CRC biomarkers. Detection of mutations in cfDNA isolated from serum and plasma has mostly concentrated in point mutations in* KRAS.* The sensitivity levels achieved using mutations in this gene were 43% at the highest with a specificity of 93% [111–113]. However, the diagnostic usefulness of tests based on the detection of tumor-derived DNA mutations is unclear since mutation status of the tumor has to be known beforehand. Circulating DNA methylation biomarkers have also been evaluated. The use of the individual methylation status of* Vimentin, NGFR, SEPT9, *and* TMEFF2 *had sensitivities ranging from 48–72% and specificities from 69-93% [114–116]. Other genes evaluated as methylation markers include* p16, APC, hMLH1, HLTF,* and* DAPK* [117]. A PCR-based assay to identify* SEPT9* methylation has been developed as an automated, commercially available kit, (Epi proColon Early Detection Assay, Epigenomics, Germany) which reportedly detects CRC with 70% sensitivity and 90% specificity [118]. Although available in Europe, it is still unavailable in Canada or the US. In a genome-scale plasma methylation marker study, two new biomarker candidates (THBD and C9orf50) with a high sensitivity and specificity for early colorectal cancer detection were identified [119]. Further studies are warranted to validate these candidate markers as CRC diagnostic tools. Furthermore, additional research efforts aimed at determining if combinations of the most promising candidate circulating methylation markers would be necessary to determine if panels, rather than single methylation markers, can improve sensitivity and specificity for CRC. Currently, the diagnostic, predictive, prognostic, and surveillance values of mutations and/or epimutations are unclear. Further analyses are needed to evaluate the CRC screening utility and clinical relevance of a blood test based on the detection of tumor-derived DNA.

#### 4.2.4. Detection of CRC Tumor mRNA in Circulation

Most investigations have focused on detecting* CEA*,* CK19,* and* CK20* transcripts. Varying degrees of sensitivities and specificities were achieved for each transcript individually, but none were optimal for the development of a CRC detection method. The combination of these markers showed a slight improvement in CRC specificity (60%–89%) and sensitivity (78%–100) [120–124], but the fact that these transcripts can be detected in patients with IBD and other malignancies, makes them unsuitable as CRC screening biomarkers. Some of the other gene transcripts that have also been studied include* TERT*,* GCC*,* MAGEA, TS, CGM2,* and* L6*, of which only GCC and* L6* showed sensitivity higher than 80% and specificity greater than 95% [125–130]. The candidate circulating CRC-specific transcripts need to be further validated in large, randomized trials to determine their clinical utility as CRC screening tests.

#### 4.2.5. Noncoding RNAs in Circulation

Since the discovery of miRNAs and the association of particular miRNAs with CRC, intense research efforts have focused on the identification of CRC-specific miRNA transcripts as potential blood biomarkers. One study reported that a 69-gene miRNA signature panel in plasma could differentiate between CRC and healthy patients [131]. A small study reported that a panel of eight miRNAs (miR-532-3p, miR-331, miR-331, miR195, miR-17, miR142-3p, miR15b, miR532, and miR-652) could accurately detect polyps [132]. Another group evaluated a three-miRNA panel (miRNA 193a-3p, miR23a, and miR-338-5p) for CRC detection achieving 80% sensitivity, 84.4% specificity, and 83.3% accuracy [133]. Although promising, these screening panels need to be tested in a larger studies to determine their clinical usefulness. Other investigations have focused on individual miRNAs, such as miR-92a. Overexpression of miR-92a has been reported to detect CRC with 89% sensitivity and 70% specificity [134]. Promising results were shown for miRNA-92, which was found to be upregulated in both tumor and plasma samples from CRC patients. Plasma levels decreased after tumor resection suggesting that miRNA-92 could be developed as both a screening and monitoring tool [134]. The expression levels of miR21 were found to correlate with disease recurrence and mortality suggesting that miR21 could be evaluated as a prognostic marker in the future [135]. Evaluation of new miRNA candidate markers, both individually and in panels, in large independent studies is necessary to determine if miRNA markers can be implemented as screening, diagnostic, and/or prognostic tools in the future.

#### 4.2.6. Biomarker Proteins

With the limited clinical applicability of CEA and CA19-9, additional candidate proteins have been proposed as CRC diagnostic protein markers. A single protein marker, TIMP-1, has detected CRC with 42–65% sensitivity and 95% specificity [136]. It is currently being tested in a large prospective study. The detection of circulating tumor-associated autoantigens and the use of commercial protein arrays have facilitated the identification of proteins that are differentially expressed and circulating in CRC patient sera. Babel et al. [137] reported 43 proteins that could distinguish between CRC patients and healthy controls. This group developed a diagnostic ELISA using two of these proteins, MAPKAPK3 and ACVR2B, which detected CRC with a specificity of 73.9% and sensitivity of 83.3%. Three additional colon-specific antigens, CCSA-2, CCSA-3, and CCSA-4, were identified by proteomic analysis of structural proteins. These proteins show very promising results as CRC diagnostic biomarkers [138, 139]. Other putative diagnostic markers that have been evaluated are the matrix metalloproteinase 9, S100A8, and S100A9 [140, 141]. Future studies with all of the proposed protein biomarkers are needed to validate the individual or panels of proteins as clinically relevant CRC molecular diagnostic tools.

#### 4.2.7. Circulating Tumor Cells

The presence of CTCs in blood is associated with progressive or metastatic disease. With the development of more advanced detection techniques, the clinical utility of CTCs as predictive or prognostic biomarkers for CRC management shows promise. Not only was it shown that presence of CTC in blood correlates with disease state [142], but also that patients with low CTCs have more favorable median progression-free and overall survival rates when compared to patients with elevated CTC levels [143, 144]. In addition, elevated CTC levels at pre- and postoperative time points in stage III and IV CRC patients undergoing curative resection were associated with postoperative relapse [145]. One of the advantages that detection of CTCs may have is that it may be used to monitor advanced stage disease in patients who do not have measurable levels of other surveillance markers in blood, such as CEA. In 2004, the US Food and Drug Administration approved the Cellsearch System (Veridex LLC, Raritan, NJ) for CTC detection for breast cancer and subsequently, for prostate and CRC. Future research will undoubtedly shed light on other clinical applications for CTCs as a predictive, prognostic, and/or surveillance marker to help clinicians make treatment decisions, predict future metastasis, and monitor disease recurrence, respectively.

## 5. Concluding Remarks

Despite numerous significant technological and methodological advances, CRC research has not yielded a novel molecular biomarker or biomarker panel suitable for population-wide screening purposes. Future studies are warranted to settle the controversy surrounding the predictive and prognostic values of some of the currently used and proposed molecular biomarkers. The presently available and developing CRC molecular markers are summarized in Table 3. Advances in genomics, transcriptomics, and proteomics will potentially identify novel candidates directly derived from the tumor cell, which in theory should be more specific. High throughput sequencing technology will undoubtedly be instrumental in accelerating the identification of novel candidate biomarkers specific for CRC and will also lead to a more comprehensive understanding of the mechanisms contributing to colorectal carcinogenesis.

The discovery of an effective, noninvasive blood-based molecular CRC screening method that could detect early stage colorectal neoplasia would be ground breaking. Patient adherence to a blood-based screening method would likely increase, resulting in an improved patient outcomes. Molecular markers also promise to shift the field to a more individualized approach to CRC treatment by taking into account the molecular background of tumor. Ultimately, the goal is to identify clinically relevant biomarkers that are cost effective, are easily assayed in a clinical setting, and contribute to patient management decisions, resulting in direct benefits for the patient.

## Figures and Tables

**Table 1:** Key characteristics of the three major CRC pathways.

	Chromosomal instability (CIN)	Microsatellite instability (MSI)	CpG island methylation (CIMP)
Prevalence	80–85%	15–20%	Up to 20%

Molecular Events	Characterized by aneuploidy, inactivation of APC/b-catenin, clonal accumulation of genetic alterations in oncogenes and tumor suppressor genes, and allelic losses and gains [49–51]	Mutations/epimutations in the MMR genes result in extensive insertions and/or deletions in microsatellites	Hypermethylation of multiple promoter CpG island loci, such as hMLH1 [9, 52]. *BRAF* mutations [53]

Clinical features	Associated with poor prognosis [54, 55]	Associated with proximal tumor location, lower staging, high grade differentiation, and abundance of tumor infiltrating lymphocytes [56, 57]	Correlates with proximal tumor location, higher prevalence in females, and BRAF mutations [58–61]

**Table 2:** Currently used CRC screening tests.

Test	Advantages	Limitations
Colonoscopy	(i) Highest performance for CRC detection (ii) Views entire colon (iii) Can take biopsies (iv) Can remove polyps	(i) May not detect some polyps, cancer, and nonpolypoid lesions (ii) Requires sedation (iii) Requires bowel preparation (iv) May cause tearing or perforation [62]

Flexible sigmoidoscopy	(i) High performance for CRC detection in rectum and lower one-third of the colon (ii) Minimal Bowel preparation (iii) Does not require sedation (iv) Can take biopsies (v) Can remove polyps	(i) Cannot detect abnormalities in the upper part of the colon (ii) Very small risk of tearing or perforation [62]

Double-contrast barium enema	(i) High performance for CRC detection (ii) Views entire colon (iii) Does not require sedation	(i) May not detect small polyps and cancer (ii) Requires bowel preparation (iii) May indicate false-positive results (iv) Colonoscopy is needed to remove polyps or perform a biopsy if abnormalities are found

Computer tomographic colonography	(i) High performance for CRC detection (ii) Views entire colon (iii) Does not require sedation (iv) Noninvasive	(i) May not detect some polyps, cancer, and nonpolypoid lesions [63, 64] (ii) Requires bowel preparation (iii) Colonoscopy is needed to remove polyps or perform a biopsy if abnormalities are found [65]

Fecal blood tests	(i) Intermediate performance for CRC detection (ii) Noninvasive (iii) No bowel preparation (iv) Low cost	(i) Fails to detect most polyps and some cancers [66, 67] (ii) May indicate false-positive results [66, 67] (iii) Dietary restrictions may be needed (iv) Needs a confirmatory colonoscopy for positive results

Stool DNA test	(i) Intermediate performance for CRC detection (ii) Noninvasive (iii) No bowel preparation	(i) Will not detect some polyps and cancers (ii) Costly (iii) Uncertain interval for screening (iv) Needs a confirmatory colonoscopy for positive results

Based on information on colorectal cancer screening provided by the National Cancer Institute and the American Cancer Society [2].

**Table 3:** Summary of conventional and innovative CRC molecular diagnostic and screening methods.

	Sensitivity (%)	Specificity (%)	Status
Tumor			
IHC^*¥*^	83	90	In use
MSI^*¥*^	55–91	90	In use
CIMP^∗∗^	N/A	N/A	Under evaluation
Stool			
gFOBT	11–64	91–98	In use
iFOBT	56–89	91–97	In use
Vimentin^∗∗^	72.5–83	53–86.9	In use
Multitarget stool test^∗^	42.4 (advanced adenomas) 92.3 (CRC)	986.6	Clinical validation
Blood			
CEA^∗∗∗^	30–63	63–100	In use
CA 19-9^∗∗∗^	18–52	79–100	In use
TIMP-1^∗∗∗^	55	95	Clinical validation
CTCs	32–94	ND- 94	Clinical validation

^*¥*^For diagnosis of Lynch Syndrome [68]. ND: not determined; N/A: not applicable. ^∗^[69], ^∗∗^[70], and ^∗∗∗^[71].
